# Lack of Evidence for an Association between Previous HEV Genotype-3 Exposure and Glomerulonephritis in General

**DOI:** 10.3390/pathogens11010018

**Published:** 2021-12-23

**Authors:** Sven Pischke, Sarah Tamanaei, Maria Mader, Julian Schulze zur Wiesch, Christine Petersen-Benz, Munif Haddad, Marylyn M. Addo, Tilman Schmidt, Tobias B. Huber, Christian F. Krebs, Oliver M. Steinmetz, Jan E. Turner, Elion Hoxha, Thomas Horvatits

**Affiliations:** 1 Department of Internal Medicine, University Medical Center Hamburg-Eppendorf, 20251 Hamburg, Germany; sarah.tamanaei@stud.uke.uni-hamburg.de (S.T.); mari.mader@uke.de (M.M.); j.schulze-zur-wiesch@uke.de (J.S.z.W.); m.addo@uke.de (M.M.A.); t.horvatits@uke.de (T.H.); 2 German Center for Infection Research (DZIF), Hamburg-Lübeck-BorstelPartner Sites, 20251 Hamburg, Germany; 3 Geschäftsbereich Zentrales Controlling, University Medical Center Hamburg-Eppendorf, 20251 Hamburg, Germany; c.petersen-benz@uke.de; 4 Institute of Clinical Chemistry and Laboratory Medicine, University Medical Center Eppendorf, 20251 Hamburg, Germany; m.haddad@uke.de; 5 III Department of Medicine, University Medical Center Hamburg-Eppendorf, 20251 Hamburg, Germany; til.schmidt@uke.de (T.S.); t.huber@uke.de (T.B.H.); c.krebs@uke.de (C.F.K.); osteinmetz@uke.de (O.M.S.); j.turner@uke.de (J.E.T.); e.hoxha@uke.de (E.H.); 6 Division of Translational Immunology, University Medical Center Hamburg-Eppendorf, 20251 Hamburg, Germany

**Keywords:** hepatitis E, glomerulonephritis, HEV, serology, extrahepatic

## Abstract

Among numerous other immune-mediated diseases, glomerulonephritis has also been suspected to be an extrahepatic manifestation of HEV infection. In this prospective study, we tested 108 patients with glomerulonephritis and 108 age- and sex-matched healthy controls at the University Hospital Hamburg Eppendorf, Hamburg, Germany, for anti-HEV IgG (Wantai test) as a marker for previous HEV exposure. A total of 24 patients (22%) tested positive for anti-HEV IgG. Males tended to be more frequently anti-HEV IgG positive (29%) in comparison to females (16%). However, this does not reach statistical significance (*p* = 0.07). Anti-HEV IgG positive patients were older in comparison to negative patients (mean 53 vs. 45 years, *p* = 0.05). The kidney function seems to be slightly decreased in anti-HEV IgG positive patients in comparison to and anti-HEV IgG negative patients basing on creatinine (*p* = 0.04) and glomerular filtration rate (GFR) (*p* = 0.05). Slightly higher values of bilirubin could be found in IgG positive patients (*p* = 0.04). Anti-HEV-IgG seropositivity rate (22%) in glomerulonephritis patients, did not differ significantly in comparison to an age- and sex-matched control cohort of healthy blood donors (31/108 positive, 29%). A total of 2/2 patients with membranoproliferative glomerulonephritis (MPGN) tested anti-HEV IgG positive (*p* = 0.002 in comparison to glomerulonephritis patients with other subtypes). In conclusion, our findings indicate that previous HEV exposure in a region where GT3 is endemic is not associated with glomerulonephritis in general. However, the subgroup of MPGN patients should be investigated in future studies. Furthermore, future studies are needed to investigate whether the observed association between anti-HEV IgG positivity and reduced GFR in glomerulonephritis patients is HEV associated or is an age-related effect.

## 1. Introduction

Hepatitis E virus (HEV) infections have been shown to be associated with a large variety of assumed extrahepatic manifestations. Particularly immunological and neurological disorders, but also renal affection has been assumed to be caused or triggered by HEV infections. However, a scientifical proof of a causal relationship is still lacking. 

One of these diseases, which has been associated with HEV infections and has been assumed to be an extrahepatic manifestation is glomerulonephritis. Glomerulonephritis is a renal disease caused by inflammatory autoimmune processes. The typical clinical signs of glomerulonephritis are hematuria, proteinuria, impaired urine output, and a reduced glomerular filtration rate (GFR) [1,2]. Glomerulonephritis has been shown to be potentially triggered by hepatitis C or B virus infections and is sometimes associated with cryoglobulinemia, a distinct immunological phenomenon [3]. In HCV- or HBV-induced glomerulonephritis deposits of immune complexes consisting of HCV-antigen, anti-HCV-IgG antibodies and a rheumatoid factor or HBV equivalents could be found and present the pathophysiological link of virus infection and kidney disease [4]. Similar mechanisms have been hypothesized for HEV infections and renal manifestations [5]. 

Large cohort studies investigating an association between HEV infections and renal diseases are still lacking. However, recently it has been described that patients with HEV infections have a reduced kidney function in comparison to patients with hepatitis A virus infections [6]. 

In line with this study, Kamar et al. investigated renal function in a French cohort of 51 solid-organ transplants before, during and after HEV infection. The glomerular filtration rate (GFR) significantly decreased in kidney and in liver transplant recipients during the acute and chronic phases of HEV infection. In five patients, renal biopsies were performed demonstrating histological features of membranoproliferative glomerulonephritis (MPGN) in two patients, relapses of IgA-nephropathy in two further patients and signs of nephroangiosclerosis in one patient. In 8 of these 51 patients (16%), no causative reason other than hepatitis E could be found for renal impairment [7]. 

Furthermore, glomerulonephritis associated with HEV infections has mainly been published in cases of European HEV genotype 3 infections. Only one single case of thrombocytopenia and membranous nephropathy has been described in a case of acute HEV genotype 1 infection [8]. All these previous reports about a possible association between HEV infections and glomerulonephritis are based on a retrospective case series or case reports. This has never been studied in a prospective study. Based on previous reports, it is assumed that glomerulonephritis might present an extrahepatic manifestation of HEV genotype 3 but not genotype 1 infections [5], but a standardized examination of a well characterized cohort is needed to clarify this issue. 

In addition to glomerulonephritis, renal involvement of HEV infections in general has also been supported by studies investigating the urine of HEV-infected patients as well as experimentally HEV-infected monkeys and rabbits. Renal biopsies from these monkeys contained protein casts in the cavities of renal tubules and signs of severe inflammation [9]. 

Taken together, previous data suggested there might be an association between HEV infections and glomerulonephritis, although it is unclear whether they directly cause glomerulonephritis or if the presence of cryoglobulinemia is needed as a link between these two entities. However, no large cohort studies or prospective data have been published to date to support this presumed association. The aim of this prospective study is to investigate a possible association of previous HEV exposure (anti-HEV IgG positivity) with glomerulonephritis in a well-defined collective of glomerulonephritis patients.

## 2. Material and Methods

In this prospective study, all adult glomerulonephritis patients of the glomerulonephritis outpatient clinic of the University Hospital Hamburg Eppendorf between May 2021 and July 2021 have been asked to participate. A total of 108 of them gave written informed consent and were included in our study. Testing for anti-HEV IgG was performed by the Wantai ELISA (Wantai Bejing, China). Laboratory data as well as baseline characteristics, such as weight and height were assessed. The estimated glomerular filtration rate (eGFR) was calculated using MDRD equation. 

To compare the anti-HEV IgG seroprevalence rate of glomerulonephritis patients with the seroprevalence in healthy adults, we performed a matched pair control analysis (matched for age and sex). For this purpose, 108 matched pairs were formed from stored serum samples from anonymous healthy blood donors without acute HEV infection and were analyzed. 

This prospective study was reviewed and approved by the Ethics Committee of the Medical Council of Hamburg (WF-138/20). The study was performed according to the recommendations of the Declaration of Helsinki.

Statistical analysis was performed as follows: Continuous variables with a non-normal distribution were expressed as median and interquartile range (IQR). Groups were compared using the Mann–Whitney U test. Categorical variables were expressed as a number (%) and compared with Fisher’s exact test. *p* values less than 0.05 were considered statistically significant. Statistical analyses were performed using SPSS, version 21.0 (IBM Corp., Armonk, NY, USA). 

## 3. Results

A total of 108 adult patients with proven glomerulonephritis have been studied. A total of 24 patients (22%) tested positive for anti-HEV IgG. Males tended to be more frequently anti-HEV IgG positive (29%) in comparison to females (16%); however, not reaching statistical significance (*p* = 0.07). 

**Figure 1:**
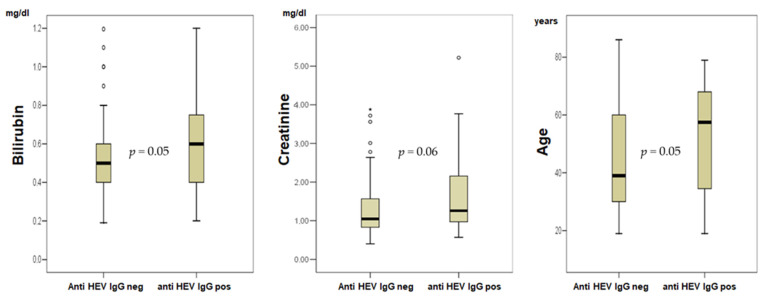
Distribution of bilirubin, creatinin and age in anti-HEV IgG positive and negative glomerulonephritis patients. *p*: *p*-Value.

Anti-HEV IgG positive patients were significantly older in comparison to negative patients (mean 53 vs. 45 years, *p* = 0.05, Figure 1). A comparison of the variables of anti-HEV IgG positive and anti-HEV IgG negative glomerulonephritis patients revealed lower values for GFR (*p* = 0.05), albumin/creatinine ratio in urine (*p* = 0.01) and protein in urine (*p* = 0.04) while significantly higher values were found for creatinine (*p* = 0.04), and bilirubin (*p* = 0.04, Figure 1). 

The anti-HEV-IgG seropositivity rate (22%) in glomerulonephritis patients, did not differ significantly in comparison to an age- and sex-matched pair control cohort of healthy blood donors (31/108 positive, 28.7%). 

Looking at the glomerulonephritis subgroups in detail, it is noticeable that 2/2 patients with MPGN (100%) were anti-HEV IgG positive, which is significantly more frequent than the anti-HEV seroprevalence in the healthy control cohort (29%, *p* = 0.002) or the rate in glomerulonephritis patients with other subtypes (22/106, *p* = 0.002). 

Detailed patients’ characteristics are shown in Table 1.

## 4. Discussion

Hepatitis E virus infections have been associated with various putative extrahepatic manifestations. While a causal relationship has been established for some of these conditions, it is still unclear whether HEV infections, similar to HBV or HCV infections, are associated with glomerulonephritis, or could even cause this immunological renal disease. Our study is the first prospective, structured assessment of anti-HEV seroprevalence in a large cohort of well-defined glomerulonephritis patients (*n* = 108). 

At first sight, the seroprevalence of 22% found here seems to be relevantly higher than the previously described seroprevalence rates of 15–17% in the German population [10,11]. However, an age- and sex-matched control cohort (*n* = 108) seems more appropriate to study this aspect, thus we identified such controls out of a cohort of 1000 blood donors. In this control cohort we found a seroprevalence of even 29%, so that glomerulonephritis patients in general cannot be considered as a risk group for previous HEV contact. These data highlight the relevance of age as risk factor for previous HEV exposure. Indeed, anti-HEV IgG positive patients in our study were older than negative patients (53 vs. 45 years, *p* = 0.05, Figure 1). This is not surprising, since with higher age and thus greater lapsed time, the risk of coming into contact with pathogens such as viruses is naturally greater than in young people. 

While our study showed no association between glomeruonephritis in general and anti-HEV IgG positivity, in the subgroup of MPGN patients 2/2 were anti-HEV IgG positive (100%). However, the subgroup of MPGN patients is very small with two patients, so this finding should not be overestimated. While for the total group of glomerulonephritis patients no significant correlation to anti-HEV seroprevalence can be found, the rare subgroup of MPGN patients needs to be re-examined in a larger follow-up study. Thus, our study showed for the first time that previous HEV contact is not a relevant trigger for the development of glomerulonephritis in general, but for the subgroup of MPGN we cannot exclude it and actually cannot make valid statements for this subgroup yet. Since MPGN is a comparatively rare disease, a multicenter study is needed.

Additionally, the current study shows that there is an association between anti-HEV IgG positivity and reduced GFR. It should be kept in mind that the number of cases in this pilot study investigating a rare disease is not large, and thus the data should not be overestimated. Nevertheless, this is the first study showing reduced GFR in anti-HEV positive patients (Figure 1). 

It has previously been shown that renal function is slightly deteriorated in the setting of acute HEV genotype 3 infection [6]. Furthermore, a complex interplay of immune cells and renal epithelium with acute kidney injury in HEV genotype 1 infections has been shown [12]. IFN-γ/chemokines and IL-18 are involved in renal damage in patients with acute HEV genotype 1 infections and thus the pathophysiological mechanism is immune modulated but not caused by renal damage by the virus itself. Unfortunately, comparable data for HEV genotype 3 still do not exist. Further pathophysiological insights and larger cohorts are needed to clarify it there is a causal relationship between previous HEV infection and impaired chronic kidney function, as demonstrated for the GFR in this present study, or if this effect is primarily caused by age. In summary, the relevance of deteriorated kidney function still stays unclear. Although cholemic nephropathy is a well described entity, we consider the borderline significant association of bilirubin and anti-HEV IgG positivity as potentially coincidental and think that this should not be overestimated. Notably, the anti-HEV IgG seroprevalence in dialysis patients has been studied by several groups, previously. However, conflicting results concerning the anti-HEV prevalence in this group of patients have been reported. A systematic review and meta-analysis, by Haffar et al., analyzed 31 studies from 17 countries [13]. The authors observed that HEV infections are more prevalent in individuals with need of dialysis, compared to those without. Of course, the limitations of a meta-analysis compared to an analysis of primary data must be considered. As none of our patients underwent dialysis, the present study does not contribute to the question of whether dialysis patients have an increased risk for HEV exposure. 

Our study has several limitations that must be taken into account. First, the studied cohort (*n* = 108 and 108 controls) is not large. However, glomerulonephritis patients are rare and thus this number of cases is adequate for a prospective monocentric observation. Second, we used the Wantai assay, and other studies used other seroassays, limiting the comparability of the data because of variability between assays. However, it should be noted that the Wantai assay is the most sensitive and well-established test in current studies. 

Summarizing, our findings from the HEV genotype 3 endemic region indicate that previous contact with HEV seems not to be a relevant trigger of glomerulonephritis in general, but can perhaps be associated with the subgroup of MPGN patients. Furthermore, our study for the first time depicts an association between anti-HEV IgG positivity and the reduction of GFR in glomerulonephritis patients. Most probably this seems to be an age-related effect.

## Figures and Tables

**Table 1:** Characteristics of anti-HEV IgG positive and negative glomerulonephritis patients.

	IgG Positive (*n* = 24)	IgG Negative (*n* = 84)	*p*-Value (Values < 0.1 Are Shown) ns = Not Significant
Sex	15 male (63%)	36 male (43%)	ns
Age	52.5(19.0)	45.2(18.2)	0.05
Type of GN			
ANCA (*n* = 28)	7 (25%)	21 (75%)	ns
IgA (*n* = 23)	6 (26%)	17 (74%)	ns
Lupus (*n* = 33)	4 (12%)	29 (88%)	ns
MCGN (*n* = 9)	2 (22%)	7 (78%)	ns
MGN (*n* = 6)	1 (17%)	5 (83%)	ns
FSGS (*n* = 3)	0	3 (100%)	ns
MPGN (*n* = 2)	2 (100%)	0	0.002
Others (*n* = 4)	2 (50%)	2 (50%)	ns
Creatinine	1.6 (1.1)	1.3 (0.8)	0.06
GFR	59.7 (34.3)	69.5 (33.0)	0.08
GPT	25.9 (17.7)	22.7 (13.3)	ns
CRP	13.8 (26.4)	5.5 (3.7)	ns
Albumin	635.8 (1371.2)	1170.4 (2378.1)	0.07
Albumin/Creatinine	696.9 (1353.9)	1261.5 (1943.1)	0.01
Protein	996.1 (2063.9)	1836.2 (3699.5)	0.03
Bilirubin	0.6 (0.3)	0.5 (0.2)	0.05
Weight	73.2 (18.4)	84.2 (27.8)	0.09
Height	173.1 (7.6)	167.9 (18.4)	ns
Immunosuppression	16 (67%)	58 (69%)	ns
Dialysis	0	0	ns
Ongoing HBV or HCV infection	0	0	ns
Previous HBV or HCV infection	0	1 (anti-HBc positive)	ns
Immunosuppressive medication:			
Prednisolon	13	38	ns
Rituximab	3	11	ns
Azathioprine	4	15	ns
Mycophenolate mofetil	5	16	ns
Quensyl	4	26	ns
Belimumab	1	2	ns
Methotrexate	1	1	ns
Cyclophosphamide	1	3	ns
Ustekinumab	1	0	ns
Tacrolimus	0	4	ns
Cyclosporin	0	2	ns
Vedolizumab	0	1	ns
